# Redescription of *Onchocerca lupi* (Spirurida: Onchocercidae) with histopathological observations

**DOI:** 10.1186/1756-3305-6-309

**Published:** 2013-10-27

**Authors:** Yasen Mutafchiev, Filipe Dantas-Torres, Alessio Giannelli, Francesca Abramo, Elias Papadopoulos, Luís Cardoso, Helder Cortes, Domenico Otranto

**Affiliations:** 1 Institute of Biodiversity and Ecosystem Research, Bulgarian Academy of Sciences, Sofia, Bulgaria; 2 Departamento de Imunologia, Centro de Pesquisas Aggeu Magalhães, Recife Pernambuco, Brazil; 3 Dipartimento di Medicina Veterinaria, Università degli Studi di Bari, Valenzano, Bari, Italy; 4 Dipartimento di Patologia Animale, Università di Pisa, Pisa, Italy; 5 Department of Infectious and Parasitic Diseases and Pathology, Faculty of School of Veterinary Medicine, Aristotle, University of Thessaloniki, Thessaloniki, Greece; 6 Department of Veterinary Sciences, School of Agrarian and Veterinary Sciences, University of Trás-os-Montes e Alto Douro, Vila Real, Portugal; 7 Parasite Disease Group, Instituto de Biologia Molecular e Celular, Universidade do Porto, Oporto, Portugal; 8 Instituto de Ciências Agrárias e Ambientais Mediterrânicas, Universidade de Évora, Évora, Portugal

**Keywords:** *Onchocerca lupi*, Vector-borne disease, Scanning electron microscopy, Light microscopy, Histology, Zoonosis

## Abstract

**Background:**

*Onchocerca lupi* is a dog parasite of increasing zoonotic concern, with new human cases diagnosed in Turkey, Tunisia, Iran, and the United States. Information about the morphology of this nematode is scant and a detailed re-description of this species is overdue. In addition, histopathological data of potential usefulness for the identification of *O. lupi* infections are provided.

**Methods:**

Male and female nematodes, collected from the connective tissue of a dog, were examined using light microscopy and scanning electron microscopy (SEM), and an histological evaluation was performed on biopsy samples from periocular tissues.

**Results:**

The morphological identification was confirmed by molecular amplification and partial sequencing of *cytochrome oxidase subunit* 1 gene. This study provides the first comprehensive morphological and morphometric description of *O. lupi* from a dog based on light microscopy, SEM, molecular characterization, and histological observations.

**Conclusions:**

Data herein presented contribute to a better understanding of this little known parasitic zoonosis, whose impact on human and animal health is still underestimated. The presence of granulomatous reactions only around the female adult suggests that the release of microfilariae from the uterus might be the cause of the inflammatory reaction observed.

## Background

The genus *Onchocerca* Diesing, 1841 (Spirurida, Onchocercidae) includes parasitic species of vast medical interest, mainly due to *Onchocerca volvulus* (Leuckart, 1893), which infects humans in tropical regions, causing severe ocular lesions, commonly referred as “river blindness” [1]. The burden of this parasite, transmitted by blackflies (Diptera, Simulidae), is severe, with 25 million people suffering from permanent visual impairment and blindness and 123 million living in areas that put them at risk of infection [2]. In addition, *Onchocerca dewittei japonica* Uni, Bain & Takaoka, 2001 [3] and *Onchocerca jakutensis* Gubanov, 1964, parasitizing wild boar in Japan and red deer in Europe and Asia respectively, have been recognized as zoonotic agents, as well [3-5]. Moreover, cases of human ocular infection have been tentatively attributed to *Onchocerca gutturosa* Neumann, 1910 and *Onchocerca cervicalis* Railliet and Henry, 1910, which primarily parasitize cattle, horses and donkeys, respectively (reviewed in [6,7]).

*Onchocerca lupi* Rodonaja, 1967 was originally described from the connective tissue of the sclera of a wolf in Georgia [8]. Recently, this parasite was reported in a human patient from Turkey who suffered from subscleral nodular lesions [9]. Following this first report, other cases of *O. lupi* infection have been increasingly diagnosed in human patients from Turkey, Tunisia [10,11], Iran [12], and the USA [7]. Ocular cases of *O. lupi* in dogs have been reported in Hungary [13], Greece [14], Germany [15], Portugal [16] and the USA [17] and in cats from the USA [18]. Although this infection is often featured by conjunctivitis, photophobia, lacrimation, ocular discharge, and exophthalmia, the first large epidemiological study carried out in dogs living in areas of Portugal and Greece, where some clinical cases were previously reported, revealed a prevalence of *O. lupi* microfilariae in up to 8.4% of the sampled animals, in spite of their apparent healthy clinical status [19].

Despite the increasingly recognised zoonotic role of this filarial worm, our knowledge about the morphology of adult nematodes is limited to studies on the type material collected from a wolf in Georgia [8,20,21], and, partially, on morphometrical data based on material collected from dogs [22], or humans [9]. Therefore, since information about the morphology of *O. lupi* is exiguous, a comprehensive re-description of this species is needed.

This study provides the first comprehensive morphological and morphometric description of *O. lupi* from a dog, based on light microscopy and scanning electron microscopy (SEM), as well as histological observations. These results are of relevance for future studies on this parasite, which is of increasing interest to human and veterinary medicine.

## Methods

Two males and one female of *O. lupi* were collected from the connective tissue of the right sclera at the necropsy of a 4-year-old female dog in the municipality of Olhão, Algarve region, southern Portugal (37°01′42″N, 7°50′33″W). The specimens were fixed and preserved in 70% ethanol. The anterior part of the female nematode and microfilariae collected from its uterus were stained with iron acetocarmine, as previously described [23]. For light microscopy observations, specimens were cleared and examined as temporary mounts in glycerine. Drawings and light microscopic images were made with a light microscope (Olympus BX51, with differential interference contrast), equipped with a drawing tube. One male and fragments of one female were dehydrated in a graded ethanol series, in order to be used for SEM observations, then sputter-coated with gold in a JEOL JFS 1200 fine coater and examined using a JEOL JSM 5510 microscope at 10 kV.

For histological examination, the entire right ocular globe and four punch biopsies (8 mm in diameter) from the right and left eyelid and bilaterally from the periocular tissues were fixed in 4% buffered formalin solution (pH 7.4), embedded in paraffin and routinely processed for light microscopy. Five μm thick sections were stained with haematoxylin and eosin before being microscopically examined.

Voucher specimens are deposited in the Helminthological Collection of the Institute of Biodiversity and Ecosystem Research, Bulgarian Academy of Sciences: N001.103, anterior and posterior fragments of one male specimen (SEM stub); N001.104, anterior extremity and fragments of mid-body of one female specimen (SEM stub); N001.105, one male specimen, one mid-body fragment of second male and fragments of single female specimen (in 70% ethanol); N001.106 and N001.107, microfilariae isolated from uteri of female nematode (microscopic slides using paraffin wax ring method).

The morphological identification was confirmed by molecular amplification and partial sequencing of *cytochrome oxidase subunit* 1 gene (*cox*1), following procedures described elsewhere [19]. Nucleotide sequences, examined by BLAST tool, showed 100% homology with the sequence from Portugal deposited in GenBank (Accession Number: KC686701 and KC686702).

## Results

### Morphological description of *Onchocerca lupi*

#### General

**Figure 1:**
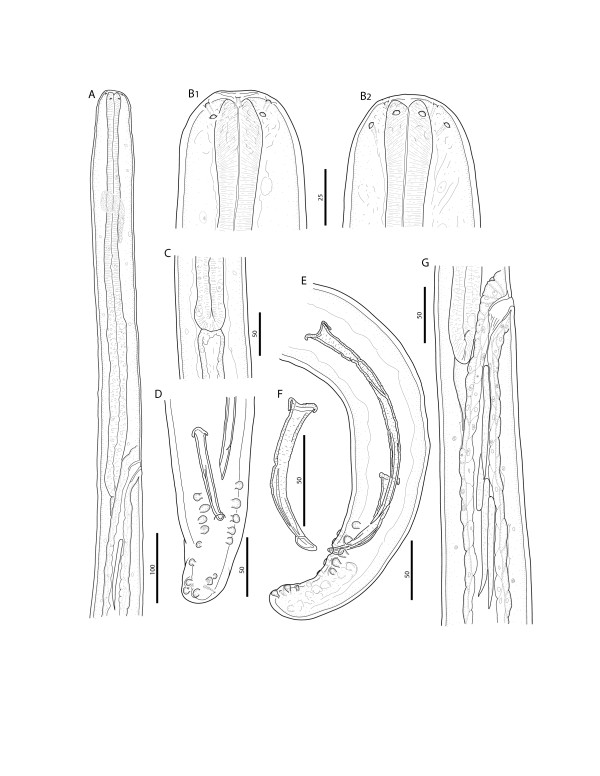
***Onchocerca lupi. *A)** Anterior part, female, lateral view. **B)** Cephalic extremity, female, lateral **(B1)** and dorsoventral view **(B2)**, respectively. **C)** Oesophago-intestinal junction, male, lateral view. **D)** Tail, male, ventral view. **E)** Posterior end, male, sinistral view. **F)** Right spicule, dextral view. **G)** Terminal part of female genital system, lateral view; note microfilariae in the ovejector. Scale-bars in micrometers.

**Figure 2:**
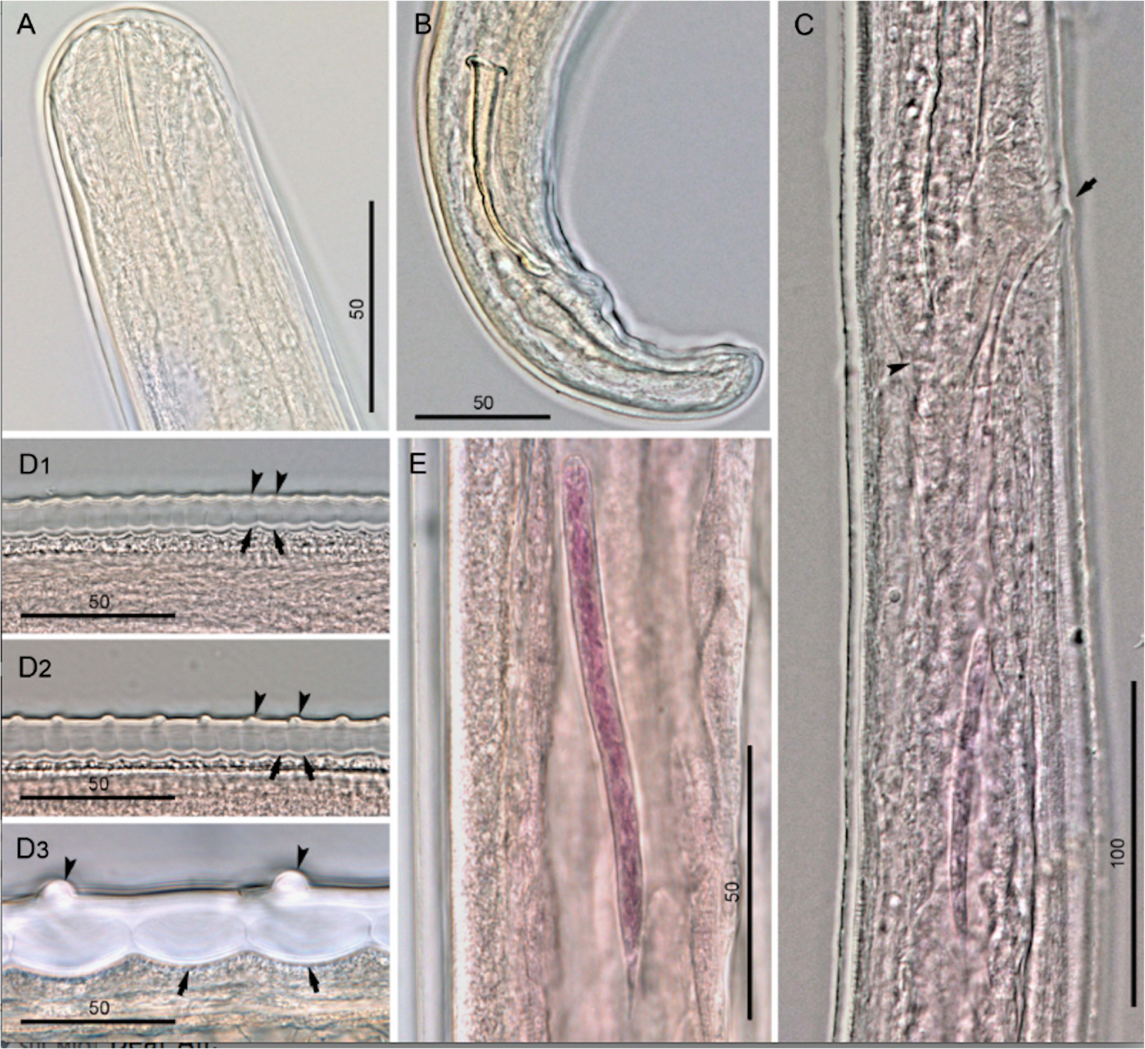
***Onchocerca lupi, *light microscopy. A)** Cephalic extremity, male, lateral view. **B)** Posterior end and right spicule, dextral view. **C)** Region of vulva, lateral view; note oesophago-intestinal junction (arrowhead) and vulva (arrow). **D1-3)** Variations in the structure of the cuticle along the body of a female: at the level of appearance of first ridges and striae **(D1)**, at the level close to the anterior body end, characterized by full formation of ridges and striae **(D2)**, and from a mid-body fragment **(D3)**; note cuticular ridges (arrowheads) and striae (arrows). **E)** Microfilaria in ovejector. Scale-bars in micrometers.

**Figure 3:**
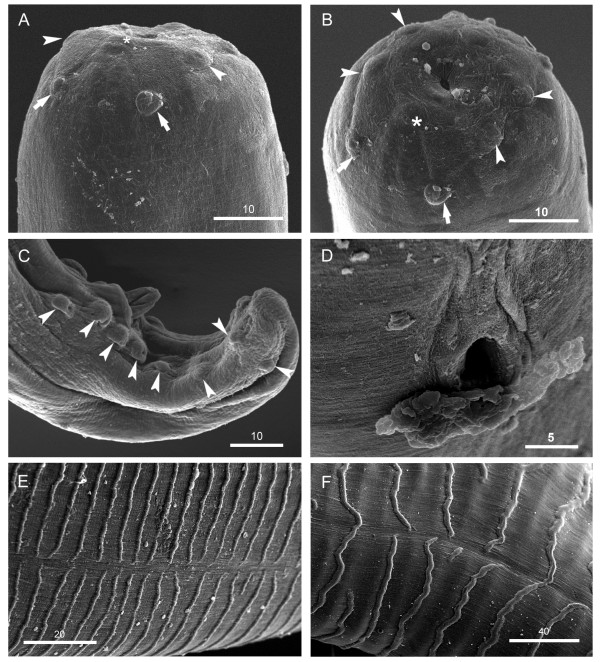
***Onchocerca lupi, *SEM. A)** Cephalic extremity, female, lateral view; note labial papillae (arrowheads), cephalic papillae (arrows) and amphid (asterisk). **B)** Cephalic extremity, female, apical view; note labial papillae (arrowheads), cephalic papillae (arrows) and amphid (asterisk). **C)** Posterior end, male, dextral view; note caudal papillae (arrowheads) and the abnormally situated eight papilla. **D)** Vulva. **E & F)** Cuticular ridges on anterior and mid-body fragments, respectively, note the interruption of cuticular ridges in the lateral fields. Scale-bars in micrometers.

Anterior end rounded, bearing four labial papillae arranged in a laterally elongated rectangle, four cephalic papillae arranged in a dorso-ventrally elongated rectangle and amphids at level of labial papillae (Figures 1B, 2A, and 3A). Mouth orifice minute (Figure 3B). Buccal cavity absent. Muscular and glandular portion of oesophagus not clearly distinct from one another (Figure 1A). Intestine narrower than glandular oesophagus (Figure 1C). Deirids and excretory pore absent.

*Male* (based on two specimens; measurements in parentheses represent those of the second specimen): distance between labial papillae in lateral and dorsoventral view 20 μm and 13 μm, respectively. Distance between cephalic papillae in lateral and dorsoventral view 14 μm and 29 μm, respectively. Body 46.9 (44.9) mm long and 114 (145) μm wide. Body width at oesophago-intestinal junction 83 (62) μm, and at level of cloaca 45 (46). Tail 79 (81) μm long, rounded. Muscular oesophagus 340 (352) μm long and 19 (15) μm wide at mid-length. Glandular oesophagus 335 (310) μm long and 36 (23) μm wide in posterior part. Nerve ring at 158 (154) μm from anterior end. Caudal alae not distinct. Caudal papillae represented by single ventral median precloacal papilla and 8 pairs of subventral papillae arranged as follow: first and second pairs precloacal, third pair adcloacal, fourth pair postcloacal, fifth pair varied in position from close to fourth pair to situated at mid-tail, last three pairs grouped near tail extremity (Figures 1D, E, and 3C). Phasmids situated between last two pairs of papillae. Left spicule 199 (201) μm long, with pointed tip (Figure 1E); spicule divided into handle 96 (88) μm and blade 103 (113) μm in length. Right spicule 91 (93) μm long, with knobbed distal end (Figures 1F, and 2B). Cuticle 10–12 μm wide, distance between cuticular striations 4 μm.

*Female* (based on single fragmented specimen): distance between labial papillae in lateral and dorsoventral view 27 μm and 13 μm, respectively. Distance between cephalic papillae in lateral and dorsoventral view 22 μm and 34 μm, respectively. Maximum body width 347 μm, width at level of oesophago-intestinal junction 71 μm. Muscular oesophagus 325 μm long and 18 μm wide at mid-length. Glandular oesophagus 314 μm long and 27 μm wide in posterior part. Nerve ring at 153 μm from anterior end. Vulva at 598 μm from anterior end (Figures 1G, 2C, and 3D). Vagina straight, 24 μm long. Ovejector straight, posteriorly directed, 3.4 mm long, with weak muscular walls. Cuticle, except extremities, composed of two distinct layers; outer layer bearing transverse ridges, often interrupted over lateral sides, and inner layer composed of bands (striae). Ridges and striae distinct at 4.9 mm from anterior body end; at this level, cuticle 6 μm thick, ridges at distance of 6–7 μm from each other, striae 6–7 μm wide (Figure 2D_1_). At about 13.0 mm from anterior body end, cuticle 10 μm thick, characterized by rounded ridges at distance of 12–14 μm from each other, striae 6–8 μm wide (Figures 2D_2,_ and 3E). In mid-body fragments, cuticle 27 μm thick, ridges at distance of up to 58 μm from each other, striae up to 32 μm wide (Figures 2D_3_ and 3F).

*Microfilariae from uteri* (metrical data are given as the range, with the mean in parentheses; n = 10): body straight to slightly bent, 105–115 (112) μm long and 6 μm wide. Anterior end rounded, containing three nuclei in the most anterior transverse row. Tail end pointed, last nucleus elongated.

### Histopathological observation

**Figure 4:**
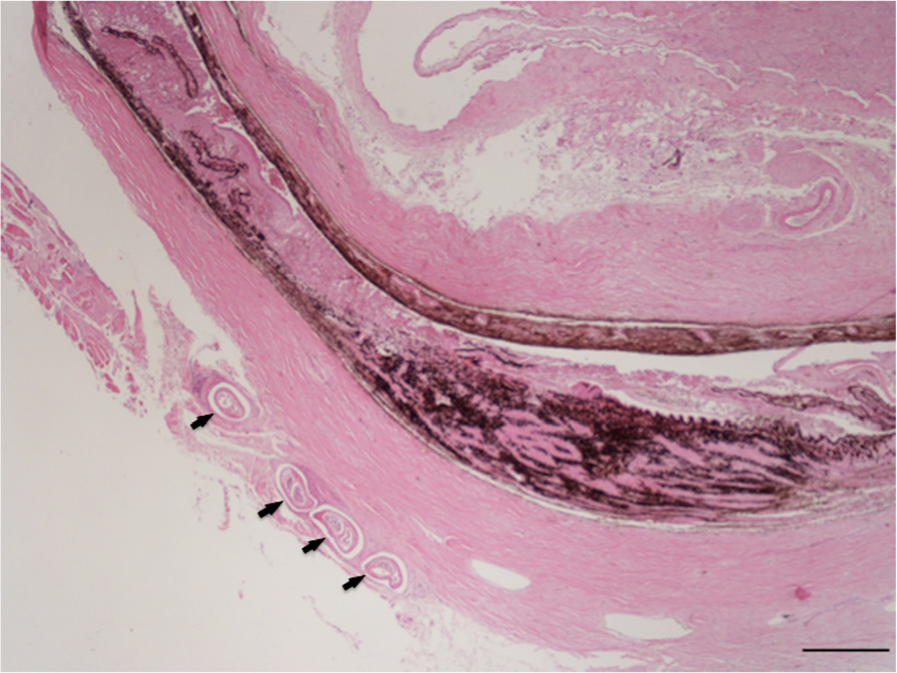
**Ocular and periocular tissue, histopathology.** Collapsed eye with four sections of *Onchocerca lupi* in the subconjunctival tissue (arrows) (H&E, scale-bar = 500 μm).

**Figure 5:**
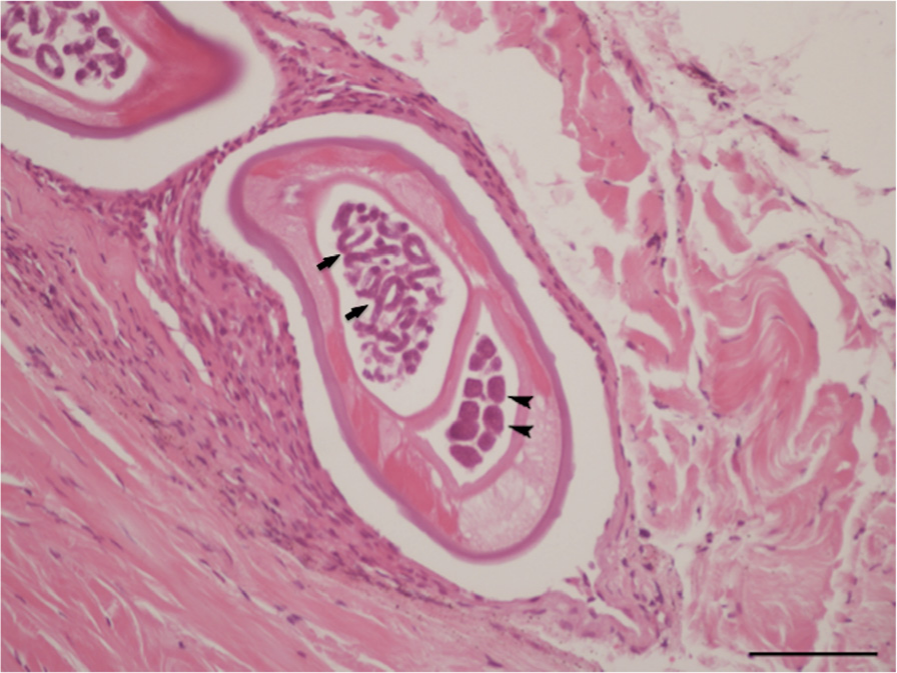
**Periocular tissue, histopathology.** In the subconjunctival sac there is a section of a coiled female of *Onchocerca lupi*, note the uteri filled with eggs containing well-developed microfilariae (arrows) or eggs in earlier stage of development (arrowheads), a faint intestine, atrophied muscle and lateral hypodermal chords (H&E, scale-bar = 100 μm).

During the histopathological examination of the conjunctival sac of the right eye, transverse to oblique sections of coiled gravid female nematodes were detected, surrounded by fibrotic tissue admixed with mononuclear cells, mainly fibroblasts and lymphocytes (Figure 4). The transverse body section of the female nematode was collapsed, measuring from 270 to 350 μm in its maximum diameter. On the sections, lateral hypodermal chords were wide, the small number of muscles cells were thick and eosinophilic. At the same level, the female body was filled with two uteri and an extremely small and thin walled intestine faintly seen in only two of the parasite sections. One of the uteri was filled with eggs containing well-developed microfilariae, while in the other uterus, eggs were at an earlier stage of development (Figure 5).

**Figure 6:**
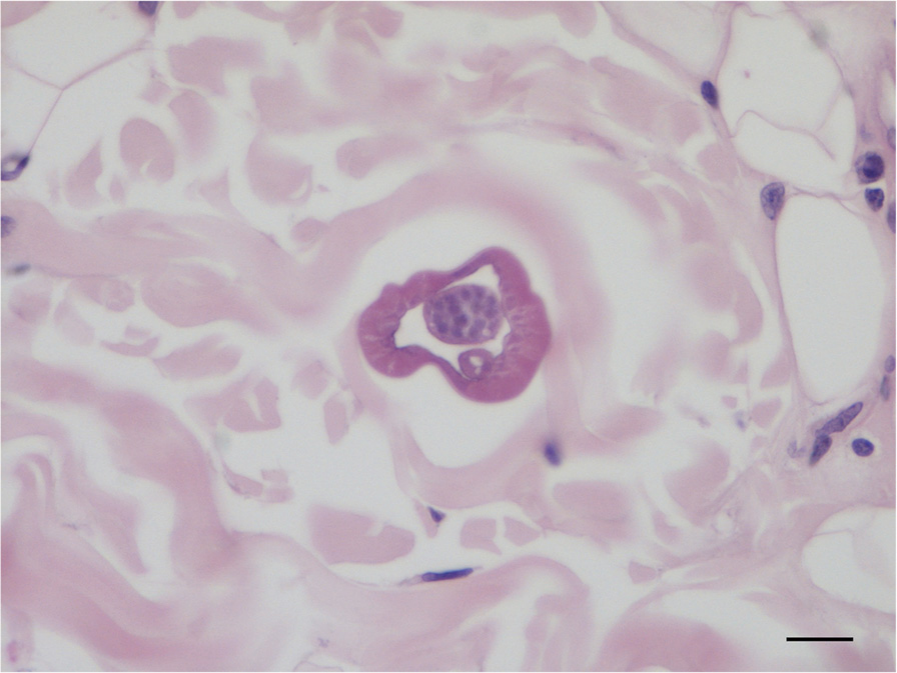
**Connective periocular tissue of a dog, histopathology.** Transverse section of an adult male of *Onchocerca lupi*, note the intestine below the vas deferens (H&E, scale-bar = 20 μm).

**Figure 7:**
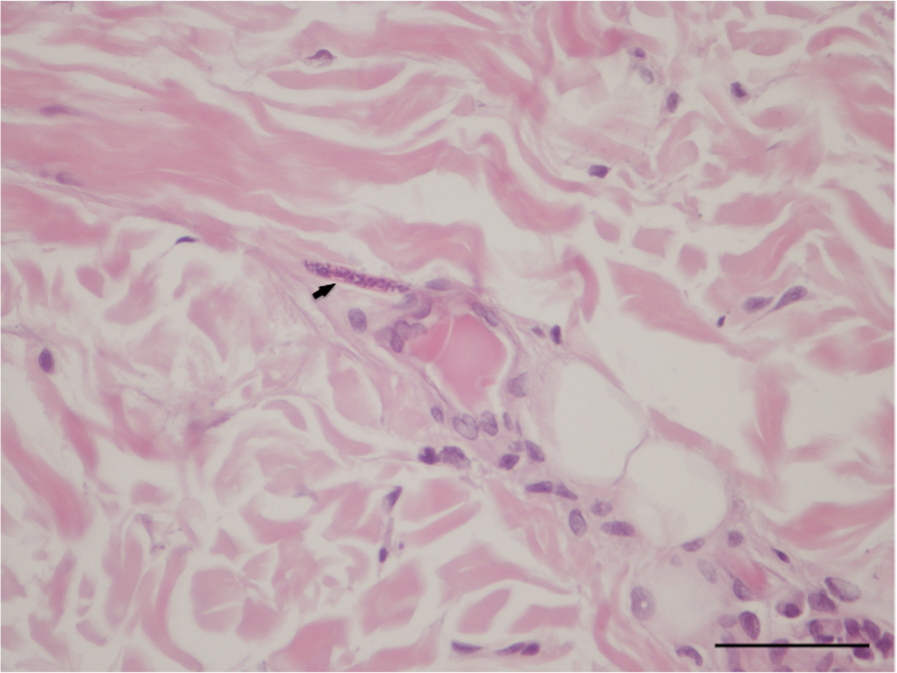
**Connective periocular tissue of a dog, histopathology.***Onchocerca lupi* microfilaria (arrow) near a small vessel (H&E, bar = 50 μm).

The dermis and the subcutis of the skin samples collected from the left and right periocular regions and eyelids were carefully checked for parasitic fragments. In the deep dermis of the right periocular region, adjacent to the superficial panniculus, a transversely sectioned parasitic body, measuring 50 μm in diameter, was located between collagen fibres. The parasitic body was characterized by an 8–10 μm thick-wall and by the presence of a thin intestine and a *vas deferens*, thus allowing it to be recognised as a male nematode (Figure 6). In the same samples, microfilariae recognised as cuticular-limited 4–5 μm wide bags of small dark nuclei, were seen in the superficial and deep dermis in the proximity of small vessels and a multifocally distributed mild perivascular inflammation was present, characterized by eosinophils and a few lymphocytes (Figure 7).

A few slender microfilariae were unevenly distributed in the perifollicular and interfollicular areas and in the deep dermis in the connective tissue among fibres, in the skin collected from the left periocular region. In this section inflammatory changes were characterized by hyperplasia and ortokeratotic hyperkeratosis with coccoid bacteria between corneocytes and chronic superficial and periadenexal perivascular infiltrate composed of lymphocytes, plasmacells, mast cells, histiocytes, neutrophils and a few eosinophils. In the skin sampled from the eyelids, microfilariae were not detected.

## Discussion

The morphology of specimens from the dog in Portugal resembles that of the type material of *O. lupi*, as initially described by Rodonaja [8] and subsequently re-described by Demiaszkiewicz and Matsaberidze [20], and Demiaszkiewicz and collaborators [21]. These similarities include the body dimensions and the length of the oesophagus of males (662–675 *vs* 600–610 μm), the morphology of spicules, the position of the vulva to the oesophago-intestinal junction, the structure of the cuticle in females, as well as the morphology and the size of microfilariae (105–115 *vs* 81–105 μm). The two male specimens studied herein are distinct from the Georgian material by a slightly longer left spicule (199–201 *vs* 172–174 μm). The female studied has a shorter oesophagus (639 *vs* 1,000-1,200 μm), and the vulva is situated at a shorter distance from the anterior end (598 *vs* 800–1,000 μm). However, these metrical differences are within the limits of intraspecific variation known for this species [10,22]. Our SEM observations of the anterior end of the female confirm the presence of a “cuticular shield reinforcing the anterior end”, as described by Demiaszkiewicz *et al.*[21]. The number of caudal papillae in the males studied corresponds to that reported by Egyed *et al.*[22] but our observations do not confirm the presence of caudal alae, reported by Rodonaja [8] and Egyed *et al.*[22]. The present re-description of *O. lupi* provided new morphological data on the position of the cephalic papillae, the detailed structure of the oesophagus, the morphology of the terminal part of the female reproductive system and the variations in the structure of the cuticle along the body of the female. The species belonging to the genus *Onchocerca* are characterized by either an undivided or divided oesophagus [24]. While previous studies did not describe the oesophagus of *O. lupi* as divided into portions [8,9,20], we succeeded in defining the border between the muscular and glandular portion, which is detectable, though not prominent (Figure 1A). Similar oesophagus morphology has been reported for several other *Onchocerca* spp., such as *O. volvulus*, *O. dukei* Bain, Bussieras & Amégée, 1974, *O. ochengi* Bwangamoi, 1969, *O. stilesi* Eberhard, 1979, *O. alcis* Bain & Rehbinder, 1986 (see ref. [25-29]). The histology of *O. lupi* here presented fits with that of previous descriptions of the parasite in dogs and humans [8,9,30]. Salient features for histological identification of the parasite at genus level where represented by the cuticular longitudinal annulation along the parasite body, detectable at the oblique sections, the atrophied muscle was replaced by hypodermal tissue, and a very thin faintly visible intestine. Both female and male adults were found coiled in the connective periocular tissue. The female was easily detected on the histological preparations, because of its large size, whereas the slender and shorter males were seen only during a thorough observation. Interestingly, the detection of the male is even more difficult due to the absence of any inflammatory reaction around the body. Conversely, the presence of a granulomatous reaction only around the female adult may be due to the release of microfilariae from the uterus, which ultimately might be the cause of the inflammatory response observed during *O. lupi* infection. The coiled gravid nematode identified by histology was encircled in a granulomatous nodule, associated with a scant mononuclear infiltrate and a fibrotic reaction, thus indicating a chronic stage of the infection. Indeed, acute inflammation is usually associated with the retrieval of microfilariae evoking an eosinophilic or lymphocytic dermatitis. In the case herein described, a few eosinophils and some other mononuclear cells were seen perivascularly close to sparse dermal microfilariae. Since the dog was from a shelter and did not receive any endo- or ecto-parasitic treatment, or was clinically assessed for possible allergy, other eosinophilic diseases could not be ruled out. It would therefore be rather difficult to determine the exact role of microfilariae in inducing skin lesions, since eosinophils were present in the deep dermal plexus as well as in the superficial ones, as expected in the case of allergic or parasitic diseases.

## Conclusions

Data herein reported contribute to a better understanding of this little known parasitic zoonotic disease, whose impact on human and animal health is still underestimated [31]. Finally, the development of serological tests for the diagnosis of canine onchocercosis would assist in a better appreciation of its real prevalence in dog populations and in the assessment of the zoonotic risk for humans living in the same areas.

## Competing interests

The authors declare that they have no competing interests.

## Authors’ contributions

YM and DO conceived the research and wrote the first draft. YM performed the morphological study and contributed to data analysis and interpretation. FA contributed the histological examination of samples. DO, YM, FD-T, LC, EP, HC, and AG sampled animals and examined skin sediments. All the authors read and approved the final version of the manuscript, contributed to the interpretation and revision of the manuscript.
